# Opportunities and Challenges in the Diagnosis and Treatment of Disorders of Consciousness

**DOI:** 10.3390/brainsci15050487

**Published:** 2025-05-06

**Authors:** Yang Bai

**Affiliations:** Affiliated Rehabilitation Hospital, Jiangxi Medical College, Nanchang University, Nanchang 330006, China; baiyang_nanchang@163.com

Disorders of consciousness (DOCs) are a dynamic and challenging field, presenting significant difficulties for clinicians and neurorehabilitation specialists due to the lack of reliable assessment methods and effective intervention strategies. Meanwhile, the mechanisms underlying consciousness recovery remain unclear, and the high heterogeneity in patient outcomes further exacerbates the long-term burden on patients’ families and the healthcare system. In recent years, the integration of neuroimaging techniques, EEG decoding, and artificial intelligence has opened new avenues for overcoming the limitations of traditional behavioral assessments [1,2,3], elucidating the dynamic characteristics of consciousness-related neural circuits, and developing individualized neuromodulation strategies [4,5].

The main aims of this Special Issue, titled “Opportunities and challenges in the diagnosis and treatment of disorders of consciousness”, were to collect clinical articles about the assessment, treatment, and prognosis of patients with DOC. As a result, nine research articles were published in this Special Issue.

Currently, bedside behavioral assessment is a common method for diagnosing the level of consciousness in DOC patients. However, evidence indicates that the misdiagnosis rate of behavioral assessments can be as high as 40% [6]. To improve assessment accuracy and reduce biases caused by patient and environmental factors, Keech and colleagues developed and validated a pre-assessment checklist designed for use prior to neurobehavioral evaluations (contribution 1). Their study found that this checklist effectively enhances interdisciplinary clinicians’ preparation for optimizing patient and environmental conditions, thereby improving the effectiveness of neurobehavioral assessments.

With the advancement of neuroimaging technologies, scientists are continually exploring more precise methods for assessing consciousness, aiming to capture subtle signs of consciousness in patients [7,8,9]. An accurate early diagnosis of patients with disorders of consciousness is crucial for formulating subsequent treatment strategies [10]. The current issue includes two articles related to neuroimaging assessments of consciousness levels. The study by Herrera-Diaz introduces multivariable pattern analysis (MVPA) to examine the periodic fluctuations of mismatch negativity (MMN) and P3a components (contribution 2). This method allows for the accurate decoding of brain responses in comatose patients through a single EEG assessment. It reveals that the MMN response in comatose patients exhibits periodic changes over time, providing valuable insights for the management and clinical decision-making regarding comatose patients. In another EEG study, Wang proposed a novel multi-task pre-trained Transformer model (MutaPT) for classifying the EEG signals of different consciousness states (contribution 3). The study found that MutaPT significantly enhanced classification performance through multi-task learning and dataset diversification, overcoming the challenges posed by limited datasets and demonstrating its potential for practical applications.

The aforementioned studies primarily focus on the assessment of consciousness in adult patients with DOC. To investigate whether existing adult assessment tools are applicable to children, particularly younger children, Colombo validated the Italian Coma Recovery Scale for Pediatrics (CRS-P) in typically developing children (contribution 4). They found that the scale may not be suitable for younger children and proposed recommendations for modifying the scale.

In terms of treatment, Qin et al. explored the impact of propofol anesthesia on brain activity in patients with vegetative state/unresponsive wakefulness syndrome (VS/UWS) during spinal cord stimulation implantation (contribution 5). The study showed that low-dose propofol anesthesia induction could induce significant changes in the EEG of VS/UWS patients, providing a reference for optimizing anesthesia management in such patients. Transcranial direct current stimulation (tDCS) has already been proven effective in treating disorders of consciousness, with scientists exploring various effective stimulation sites, including the dorsolateral prefrontal cortex, primary motor cortex, and parietal cortex [11,12,13,14,15]. Wan’s study compared the neuroregulatory effects of frontal and parietal tDCS on DOC patients (contribution 6). The results showed no significant differences in the neurobehavioral improvement between the two, while EEG data suggested potential differences in their neuroregulatory mechanisms.

A key issue in the neurorehabilitation process for patients with acute brain injury is how to establish personalized care goals. Rodriguez Mateos investigated the impact of the life history questionnaire on the formulation of personalized care goals for patients with clinical cognitive motor dissociation in an acute neurorehabilitation unit (contribution 7). The study found that the life history questionnaire played a significant role in developing personalized care goals within the “Activities and Participation” category of the ICF framework.

Ischemic–hypoxic brain injury following cardiac arrest is one of the major causes of coma in patients. Accurately predicting neurological recovery is critical for guiding clinical treatment. Shivji conducted a retrospective study to analyze the EEG characteristics of patients admitted to the ICU after cardiac arrest (contribution 8). The study found that high-malignancy EEG, seizures, and myoclonus were associated with poor patient prognosis, while patients with benign EEG patterns exhibited better outcomes. This suggests that dynamic changes in EEG signals could provide important insights into clinical decision-making.

Delayed cerebral ischemia (DCI) is one of the most common and fatal complications of acute subarachnoid hemorrhage, and an early prediction of DCI risk is of significant importance. Deininger investigated the relationship between glucose index and the occurrence of DCI (contribution 9). This study found that patients’ glucose levels significantly increased prior to the onset of DCI and further escalated after its occurrence, suggesting that glucose management may play a crucial role in the prevention and treatment of DCI.

This Special Issue compiles several important research findings on the diagnosis and treatment of DOC, highlighting significant advancements in the field regarding assessment tools, therapeutic approaches, and prognostic predictions. From behavioral assessments to the application of neuroimaging technologies, from the exploration of novel neuromodulation techniques to the development of personalized care goals, these studies offer new insights and methodologies for improving the diagnosis, treatment, and care of DOC patients.

Future research directions should continue to focus on the following areas: First, as neuroimaging technology continues to advance, efforts should be made to develop multimodal assessment systems that integrate EEG, neuroimaging, and behavioral data to create a dynamic consciousness monitoring system. Second, building on existing neuromodulation techniques, the further exploration of non-invasive, personalized neuromodulation methods is needed, along with the integration of real-time neural feedback to tailor stimulation parameters. Additionally, multi-parameter joint prediction models could be developed to improve the accuracy of neurofunctional prognosis prediction and promote the implementation of preventive treatment strategies. Finally, there should be a stronger emphasis on cross-disciplinary collaboration between neuroscience, engineering, and clinical medicine to establish a full-cycle care system based on the ICF framework. In conclusion, with the ongoing deepening of multidisciplinary collaboration and technological innovations, future research will open new avenues in the fields of consciousness decoding, neurorepair, and precision medicine, driving the diagnosis and treatment of disorders of consciousness toward more precise, personalized, and diversified approaches.
